# Reinterpreting Ethnic Patterns among White and African American Men Who Inject Heroin: A Social Science of Medicine Approach

**DOI:** 10.1371/journal.pmed.0030452

**Published:** 2006-10-24

**Authors:** Philippe Bourgois, Alexis Martinez, Alex Kral, Brian R Edlin, Jeff Schonberg, Dan Ciccarone

**Affiliations:** 1 Department of Anthropology, History and Social Medicine, University of California San Francisco, San Francisco, California, United States of America; 2 Center for AIDS Prevention Studies, University of California San Francisco, San Francisco, California, United States of America; 3 RTI International, San Francisco, California, United States of America; 4 Center for the Study of Hepatitis C, Division of Gastroenterology and Hepatology, Weill Medical College of Cornell University, New York, New York, United States of America; 5 Department of Family and Community Medicine, University of California San Francisco, San Francisco, California, United States of America; University of Colorado, United States of America

## Abstract

**Background:**

Street-based heroin injectors represent an especially vulnerable population group subject to negative health outcomes and social stigma. Effective clinical treatment and public health intervention for this population requires an understanding of their cultural environment and experiences. Social science theory and methods offer tools to understand the reasons for economic and ethnic disparities that cause individual suffering and stress at the institutional level.

**Methods and Findings:**

We used a cross-methodological approach that incorporated quantitative, clinical, and ethnographic data collected by two contemporaneous long-term San Francisco studies, one epidemiological and one ethnographic, to explore the impact of ethnicity on street-based heroin-injecting men 45 years of age or older who were self-identified as either African American or white. We triangulated our ethnographic findings by statistically examining 14 relevant epidemiological variables stratified by median age and ethnicity. We observed significant differences in social practices between self-identified African Americans and whites in our ethnographic social network sample with respect to patterns of (1) drug consumption; (2) income generation; (3) social and institutional relationships; and (4) personal health and hygiene. African Americans and whites tended to experience different structural relationships to their shared condition of addiction and poverty. Specifically, this generation of San Francisco injectors grew up as the children of poor rural to urban immigrants in an era (the late 1960s through 1970s) when industrial jobs disappeared and heroin became fashionable. This was also when violent segregated inner city youth gangs proliferated and the federal government initiated its “War on Drugs.” African Americans had earlier and more negative contact with law enforcement but maintained long-term ties with their extended families. Most of the whites were expelled from their families when they began engaging in drug-related crime. These historical-structural conditions generated distinct presentations of self. Whites styled themselves as outcasts, defeated by addiction. They professed to be injecting heroin to stave off “dopesickness” rather than to seek pleasure. African Americans, in contrast, cast their physical addiction as an oppositional pursuit of autonomy and pleasure. They considered themselves to be professional outlaws and rejected any appearance of abjection. Many, but not all, of these ethnographic findings were corroborated by our epidemiological data, highlighting the variability of behaviors within ethnic categories.

**Conclusions:**

Bringing quantitative and qualitative methodologies and perspectives into a collaborative dialog among cross-disciplinary researchers highlights the fact that clinical practice must go beyond simple racial or cultural categories. A clinical social science approach provides insights into how sociocultural processes are mediated by historically rooted and institutionally enforced power relations. Recognizing the logical underpinnings of ethnically specific behavioral patterns of street-based injectors is the foundation for cultural competence and for successful clinical relationships. It reduces the risk of suboptimal medical care for an exceptionally vulnerable and challenging patient population. Social science approaches can also help explain larger-scale patterns of health disparities; inform new approaches to structural and institutional-level public health initiatives; and enable clinicians to take more leadership in changing public policies that have negative health consequences.

## Abstract 2

Bourgois and colleagues found that the African American and white men in their study had a different pattern of drug use and risk behaviors, adopted different strategies for survival, and had different personal histories.

## Editors' Summary

**Background.:**

There are stark differences in the health of different ethnic groups in America. For example, the life expectancy for white men is 75.4 years, but it is only 69.2 years for African-American men. The reasons behind these disparities are unclear, though there are several possible explanations. Perhaps, for example, different ethnic groups are treated differently by health professionals (with some groups receiving poorer quality health care). Or maybe the health disparities are due to differences across ethnic groups in income level (we know that richer people are healthier). These disparities are likely to persist unless we gain a better understanding of how they arise.

**Why Was This Study Done?:**

The researchers wanted to study the health of a very vulnerable community of people: heroin users living on the streets in the San Francisco Bay Area. The health status of this community is extremely poor, and its members are highly stigmatized—including by health professionals themselves. The researchers wanted to know whether African American men and white men who live on the streets have a different pattern of drug use, whether they adopt varying strategies for survival, and whether they have different personal histories. Knowledge of such differences would help the health community to provide more tailored and culturally appropriate interventions. Physicians, nurses, and social workers often treat street-based drug users, especially in emergency rooms and free clinics. These health professionals regularly report that their interactions with street-based drug users are frustrating and confrontational. The researchers hoped that their study would help these professionals to have a better understanding of the cultural backgrounds and motivations of their drug-using patients.

**What Did the Researchers Do and Find?:**

Over the course of six years, the researchers directly observed about 70 men living on the streets who injected heroin as they went about their usual lives (this type of research is called “participant observation”). The researchers specifically looked to see whether there were differences between the white and African American men. All the men gave their consent to be studied in this way and to be photographed. The researchers also studied a database of interviews with almost 7,000 injection drug users conducted over five years, drawing out the data on differences between white and African men. The researchers found that the white men were more likely to supplement their heroin use with inexpensive fortified wine, while African American men were more likely to supplement heroin with crack. Most of the white men were expelled from their families when they began engaging in drug-related crime, and these men tended to consider themselves as destitute outcasts. African American men had earlier and more negative contact with law enforcement but maintained long-term ties with their extended families, and these men tended to consider themselves as professional outlaws. The white men persevered less in attempting to find a vein in which to inject heroin, and so were more likely to inject the drug directly under the skin—this meant that they were more likely to suffer from skin abscesses. The white men generated most of their income from panhandling (begging for money), while the African American men generated most of their income through petty crime and/or through offering services such as washing car windows at gas stations.

**What Do These Findings Mean?:**

Among street-based heroin users, there are important differences between white men and African American men in the type of drugs used, the method of drug use, their social backgrounds, the way in which they identify themselves, and the health risks that they take. By understanding these differences, health professionals should be better placed to provide tailored and appropriate care when these men present to clinics and emergency rooms. As the researchers say, “understanding of different ethnic populations of drug injectors may reduce difficult clinical interactions and resultant physician frustration while improving patient access and adherence to care.” One limitation of this study is that the researchers studied one specific community in one particular area of the US—so we should not assume that their findings would apply to street-based heroin users elsewhere.

**Additional Information.:**

Please access these Web sites via the online version of this summary at http://dx.doi.org/10.1371/journal.pmed.0030452.

- The US Centers for Disease Control (CDC) has a web page on HIV prevention among injection drug users
- The World Health Organization has collected documents on reducing the risk of HIV in injection drug users and on harm reduction approaches
- The International Harm Reduction Association has information relevant to a global audience on reducing drug-related harm among individuals and communities
- US-focused information on harm reduction is available via the websites of the Harm Reduction Coalition and the Chicago Recovery Alliance
- Canada-focused information can be found at the Street Works Web site
- The Harm Reduction Journal publishes open-access articles
- The CDC has a web page on eliminating racial and ethnic health disparities
- The Drug Policy Alliance has a web page on drug policy in the United States

## Introduction

Disparities in health across ethnic and class categories are widely documented and represent an urgent problem for US society. Causal explanations for disparities, however, are still debated [1–4], especially with respect to the relative effects of class, culture, gender, and access to quality care [5–7]. The meaning and practice of “cultural competence” in medicine is debated because of the risk of stereotyping the diversity that exists within cultural groups [4,8,9]. One population group that is particularly vulnerable to negative health outcomes and social stigma is street-based substance abusers [10–12]. Using a multi-methodological approach, we document the variability and complexity, by ethnicity, of factors and behaviors salient to understanding this population group.

The National Institutes of Health (NIH) requires researchers to document “race/ethnicity” in order to document health disparities [2]. Racial categories, however, are a social construct and cannot usefully document genetic differences or cultural attributes at the level of the individual because of the greater diversity that exists within large population groups than between them [13,14] (for sociological critiques see [15–17]). Furthermore, the NIH and US Census dichotomy between ethnic and racial categories is logically inconsistent and reflects politically driven classifications rather than scientific reality [14]. Hospital-based quantitative researchers have also found that asking patients to describe their race/ethnicity in their own words was more effective for tracking health-related data than was having patients choose from a set of predetermined categories [18]. The American Anthropological Association advocates abandoning the historically fraught classification “race” to avoid its misleading implications, and instead using the more dynamic category “ethnicity” [14]. Cultural anthropologists define ethnicity as a socially contingent identity that is simultaneously imposed on individuals and is also chosen by them through social interaction in historically determined contexts [19]. Some ethnicities become “racialized”—a term describing how rigid distinctions between groups based on skin color and appearance are created through racism and economic inequality in a particular social setting and become conceptualized as irrevocable essences determined by genes and/or culture [20].

There is widespread documentation of epidemiological patterns to drug use according to ethnicity and age group [21–23], but the few theoretical explanations that exist remain speculative [24,25]. African Americans, for example, are overrepresented among the aging “baby boom” cohort of street-based heroin injectors and crack smokers who suffer increasingly complicated medical conditions, but they are relatively underrepresented among youth injectors [10,11,26–28]. A theoretical understanding of drug use as a changing phenomenon shaped by history and social structure could facilitate the development of systematic, large-scale health services delivery interventions and public health initiatives. At the clinical level, street-based drug injectors often frustrate practitioners and are costly to institutions [10,11,29,30].

### Aims of This Study

We proposed to build an explanation of different patterns of drug use and survival strategies between African American and white street-based heroin injectors through a social science-based understanding of the large-scale institutional power constraints that shape individual behavior. Our approach involved a close examination of the distinct experiences of street-based illegal drug use among African Americans and whites in San Francisco, informed by cross-methodological dialog. Our goal was to elucidate how a basic science of social medicine might contribute to improvements in clinical cultural competence toward socially vulnerable patients without reducing their characteristics and diversity to a static list of self-destructive behaviors or self-fulfilling labels. By understanding the social, institutional, and historical forces that lead to negative health behaviors we hoped to contribute to formulating targeted, pragmatic prevention messages in public health [31] and to developing institutional and policy-level interventions (for examples, see [32–34]).

## Methods

Our data derive from a cooperative multidisciplinary effort of contemporaneous projects, funded by the US NIH, that includes ethnographic, epidemiological, and clinical components. The research team included an ethnographer (PB), a sociologist (AM), an epidemiologist (AK), an infectious diseases physician and epidemiologist (BRE), a photoethnographer (JS), and a physician trained in both qualitative and quantitative research methods (DC).

### Participant-Observation Ethnography

#### Data sources.

Our ethnographic database consists of: (1) 3,600 pages of field notes and transcribed interviews coded in Atlas.ti software (http://www.atlasti.com), and (2) 600 digitized images indexed in iView MediaPro (http://www.iview-multimedia.com) from our database of over 11,000 photographs taken by JS of street-based drug users in their natural context. We collected this qualitative and photographic data in one San Francisco neighborhood through participant-observation fieldwork from November 1994 to November 2004 in the injection sites and homeless encampments that anchored a social network of approximately two dozen white, Latino, and African American street-based heroin addicts. (For an example of an injection site see photo by JS at http://www.publicanthropology.org/Photogallery/B&S-Sid-in-Hole.htm.) The total membership of the group fluctuated at any given moment as individuals left or returned to the scene due to arrest, treatment, relapse, out-migration, illness, and death. Over ten years, consequently, we collected detailed field notes, tape recordings (including life histories), and photographic documentation on over 70 individuals. Approximately 20% of our research participants were Latinos and Latinas, and 10% were women.

We limit our discussion to the African American and white men in our sample because of space constraints and sample size. Furthermore, the Latino and Latina injectors drew less consistent boundaries around themselves. They sometimes shifted their ethnic identification according to nationality and length of stay in the US depending on the context of a particular social interaction. Some identified around a racialized identity and others did not.

#### Theoretical framing and ethnographic technique.

Our ethnographic research design and coding strategy was guided by a social science theoretical understanding of the link between large-scale power relations and individual risky practices that shape the spread of blood-borne disease among injectors. We focused data collection, consequently, on how ethnicity, gender, sexuality, status hierarchies, and income-generation options interface with drug consumption practices and survival strategies. Following Kleinman et al.'s [35] strategy of eliciting explanatory models (for an application see chapter 17 in [36]) to understand nonadherent patients, we conducted conversational interviews in the natural environments of street-based injectors and clinicians to assess the mechanisms that result in negative (as well as positive) interactions with medical and social services. We documented the conceptualizations of both clinical service providers and patients.

We identified and explored the vital social structural and institutional interfaces of street-based substance users with the broader society in five domains: (1) law enforcement; (2) social services; (3) medical care; (4) modalities of income generation; and (5) social support arrangements. We also observed interfaces with the general public, family members, and institutional representatives on the street, in homes, and at public offices—including the county hospital, community-based clinics, and jail. We simultaneously collected both self-report and observational qualitative data. This technique allowed us to repeatedly triangulate self-reported risk with directly observed risk-taking as it occurred in its natural context to increase accuracy, and to probe respondents' understanding of risk-taking. The ethnographers (PB and JS) spent nights in homeless encampments and accompanied individuals on their sorties in search of income and drugs.

#### Photoethnography.

Conversations and interactions were tape-recorded and/or photographed following an informed consent protocol approved by the Internal Review Board of the University of California San Francisco (see Texts S1 and S2 for sample consent forms). Photoethnographic participant-observation data collection represents a special ethical challenge [37,38] and requires the active collaboration of respondents for full access to documenting intimate daily routines on a regular basis. Photographs taken in the natural environment can powerfully supplement and triangulate with ethnographic and statistical data. Pictures document for subsequent examination social interactions and the details of material environment, technology use, and body language that are often missed by observers during the spur of the moment or are altered retrospectively when self-reported. We also gave our research participants copies of their photographs and asked for comments. In addition to allowing confirmation of permission for publication, this “photo-elicitation” method of conversational interviewing [39] generated further detailed self-report and self-reflection. (For examples see photos by JS at http://www.publicanthropology.org/Photogallery/B&S-HankwithFlag.htm and http://www.publicanthropology.org/Photogallery/B&S-Mindy&Petey.htm.)

### Epidemiological Data

Our epidemiological data draw from the Urban Health Study (UHS; University of California, San Francisco) database of over 30,000 interviews with more than 12,000 injection drug users from 37 semiannual cross-sections from 1986 to 2005 [40,41]. The UHS administered sociodemographic and behavioral face-to-face interviews and blood draws (for HIV and hepatitis C virus [HCV] testing) in single-room occupancy hotels, churches, and community-based meeting venues located in five neighborhoods in San Francisco where drug users congregate on the streets: Bayview, Mission, Tenderloin, Western Addition, and South of Market. The UHS used targeted sampling methods to recruit a community-based sample of active injectors every six months in each neighborhood [42]. Persons aged 18 years or older who had injected illicit drugs in the previous 30 days (as confirmed through visual inspection of injection stigmata) or who had previously participated in the UHS were eligible for participation. They were allowed to participate as often as every six months. Participants received pre- and post-HIV/HCV test counseling and a small stipend ($15–$20/visit).

### Clinical Ethnography

Our clinical database is primarily qualitative and observational and consists of 250 pages of field notes drawn from clinical practice (by DC) in the natural environment of the ethnographic sample and in alternative community-based service sites. In addition, in-depth qualitative interviews were conducted (by DC) with approximately 70 individuals recruited through the epidemiological study to further explore the clinical implications of our qualitative and quantitative data. Clinical inquiry focused on injection technique, including routes, sites, and skin hygiene, as related to risks for bacterial infection, for example abscess and cellulitis.

### Integrated Collaborative Study

Toward the end of this cross-methodological collaboration (2004–2005) we developed a “strategically targeted intensive case study” protocol to collect supplemental qualitative data on a dozen individuals who were not members of our original ethnographic social network sample, but who had behavioral profiles that warranted further exploration. These participants were strategically identified through the epidemiological interviews in the last rounds of the UHS cross-section and immediately referred to the ethnographic team, who then engaged in qualitative follow-up with these individuals in their natural street environment. Exploring counterfactual cases that were identified through the epidemiological infrastructure enabled a more in-depth understanding of the behaviors and characteristics identified as salient by our preliminary analysis. The strategically targeted follow-up cases represented the only new ethnographic data specifically collected through the epidemiological collaboration, and they diversified the reach of our original ethnographic sample. All the qualitative clinical data, in contrast, were collected in ongoing dialog with both the ethnographic and epidemiological projects dating back to before 2000. The clinical research informed both the ethnographic and epidemiological studies by extending and focusing ethnographic observations around medically relevant practices and contributing to questionnaire development. Clinical observations also contributed to developing hypotheses regarding modes of injection, vein scarring, and soft tissue infection.

### Analysis

The current analysis required extensive review of previously collected ethnographic material in collaborative dialog among the ethnographic, clinical, and epidemiological investigators. This dialog involved iterative analyses of the previously collected epidemiological database of street-based injectors interviewed during the period 2000–2005 in San Francisco (*n* = 6,655). First, we situated our small, purposeful ethnographic network sample demographically and behaviorally within this large UHS sample to abstract a relevant sociodemographic subsample (Table 1) that consisted of all the UHS respondents interviewed between the years 2000 and 2005 who were men reporting daily heroin injection and who identified themselves as either African American or white (*n* = 1,068). (In the larger UHS sample, 26% were women and 6.7% were Latinos or Latinas.) We then identified 14 variables on the UHS questionnaire as relevant to test the patterns identified by our ethnographic data and our clinical observations. We also stratified those variables by the ethnic categories African American and white and by the median age of UHS respondents in 2002 (45 years), which was also the age of the youngest ethnographic research participant. We stratified by age because in a separate ethnographic study among youth injectors in San Francisco [6] we found that street-based injectors maintained age-segregated social networks and tended to differentiate themselves as being members of a younger versus an older generation. We tested whether observed epidemiological differences by ethnicity were statistically significant by conducting Chi-square tests and Fisher exact tests for parametric categorical variables, and Student t-test (parametric) and Wilcoxon rank test (nonparametric) for continuous variables (Table 1).

**Table 1:**
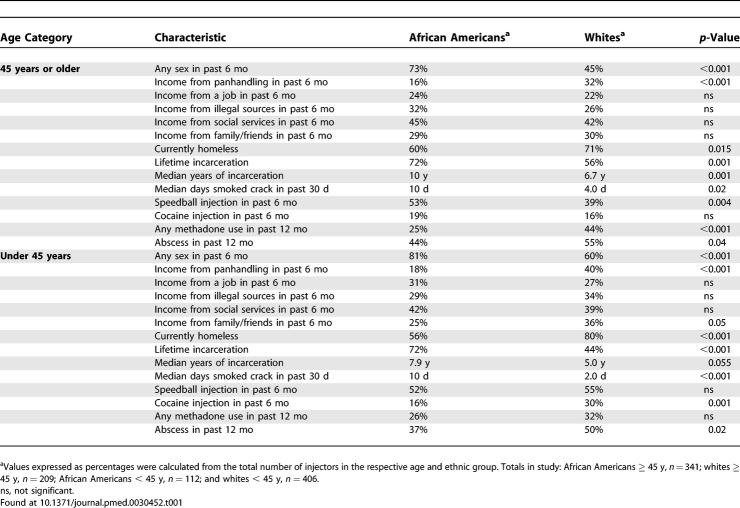
Distributions of Selected Behaviors by Age and Race among Male Heroin Injectors in San Francisco, 2000–2005 (*n* = 1,068)

Our cross-methodological quantitative/qualitative analytical dialog was purposefully iterative to synthesize hypotheses and data: Ethnographic observations generated hypotheses; hypotheses led to further empiric analyses; and data subsequently generated further hypotheses. This process enabled us to explore subtle dimensions of identity and social processes, as well as potential biases in the data due to the collection of socially desirable self-reports on the taboo subjects of drugs, sex, and crime in both our quantitative and qualitative data.

## Results

### Income-Generating Strategies and Social Interactions

Our ethnographic observations revealed antagonistic interactions between street-based African American and white heroin injectors who survived in the same public spaces. Despite routinely purchasing drugs together and sharing injection paraphernalia, they identified themselves as separate groups based on skin color. They considered this distinction to be self-evident and justified their sense of difference in terms of moral worth and personal dignity. They routinely referred to one another with racist epithets and derogatory dictums. In short, the group identities of “African American” and “white” are socially constructed, racialized ethnicities. Their sense of difference was organized through opposition to one another in everyday interactions [19].

Our ethnographic field notes, tape recordings, and photographs documented that most African American heroin injectors conceived of themselves as successful outlaws. The participants in our study generated the majority of their income through petty crime and/or through short-term service-related income generation such as washing car windows at gas stations. They considered passive panhandling to be demeaning and rejected formal day labor as exploitative. They often hid the fact that they were homeless or marginally housed. They tended to dress in up-to-date fashions and strove to maintain a public appearance of being in control of their lives. They actively maintained relationships with a wide range of acquaintances in the larger society, including their families, often visiting extended kin on holidays and birthdays. Most actively pursued romantic heterosexual relationships and asserted with pride that they were sexually active. They thought of themselves as effective professional outlaws.

The white participants, in contrast, tended to consider themselves destitute outcasts. They expressed a sense of decrepitude, passivity, and crisis. They subscribed to a medicalized conception of addiction as a disease and referred to themselves as being “sick.” Most maintained no relationship with their families and they often wore tattered clothes and appeared disheveled. Most reported erectile dysfunction without embarrassment, and claimed not to be interested in either sex or romance. Consistent with their defeated demeanor, the whites generated most of their income through passive panhandling, although they also performed day labor for local business owners on an hourly or piece-rate basis, often at below minimum wage. They referred to themselves as homeless and publicly projected that assertion (even when they had access to housing) to solicit help.

All of these ethnic distinctions were documented in hundreds of coded entries from our notes, transcripts, and photographs, and the patterns repeated themselves with relatively few exceptions to the point of saturation. Furthermore, most of the inconsistencies that we documented in these patterns were either expected because of context, or were noted as being anomalous by respondents on the street.

Seven questions from the epidemiological survey directly addressed specific components of the overall ethnic personae outlined above: Among heroin injectors 45 years and older, African Americans were less likely than whites to report that they were “homeless” (60% versus 72%; *p* = 0.006). African Americans were more likely to report having “any sex with women in the past 6 months” than whites (73% versus 47%; *p* < 0.001) and less likely to report receiving income from panhandling (17% versus 33%; *p* < 0.001). All of these quantitative associations supported the ethnographic findings. There were no significant differences between the two groups, however, for income generated from legal employment, illegal sources, social services, or family friends (Table 1). All of these comparative ethnic patterns also held among the under-45-year-old injectors (Table 1).

### Childhood Socialization and Institutional Interactions

Most of the injectors in the ethnographic network grew up in San Francisco in impoverished households. The childhood trajectories of the African Americans and the whites, however, differed. African American male injectors followed distinct adolescent paths into careers of long-term heroin addiction and unemployment in a historical era of inner-city deindustrialization [43–46] that coincided with the initiation of the “War on Drugs” by President Nixon in 1971. Our life history tape recordings revealed that identifiable institutional and social structural forces shaped a more outlaw-identified street-based persona among young African Americans than among young whites. Consistent with findings reported in the social science literature on the California economy [44,47,48], most of the African Americans reported that their fathers were laid off from manual labor jobs in San Francisco when industrial work disappeared. Many explained that their parents had emigrated from the Deep South to find work in the San Francisco shipyards during World War II. They referred explicitly to the poverty and racism that prevailed in their parents' communities of origin. Most had visited their parents' hometowns and they still occasionally attended regional family reunions. All of the African Americans reported juvenile incarceration for gang activity during their teenage years before they started injecting heroin.

Many of the whites also referred to the poor, rural origin of their parents, but few knew the details of their home communities. Most were employed as adolescents in the small businesses established by their parents after immigrating to San Francisco (e.g., sign painting, bartending, or foundry work). Only one of the whites reported former membership in a teenage gang. He qualified that self-report, however, by laughing at his ethnic exceptionalism, “I was the only white boy living in the projects and I was the only white in the Medallions [gang].” The whites, consequently, were generally not incarcerated until they engaged in crime associated with their physical dependence on heroin in their early 20s.

Almost all of the street-based injectors in our ethnographic sample were routinely searched, evicted, and ticketed by the police and occasionally arrested. Law enforcement activities sometimes directly interfered (occasionally purposefully) with accessing medical and public health services. On several occasions, for example, the police confiscated prescribed medications and bandage supplies. Possession of syringes (obtained at the legal, public health-funded needle exchange program) was the most frequent precipitating factor for arrest or ticketing among both African Americans and whites. (Possession of syringes without a prescription for needle exchange attendees in San Francisco was illegal until 2005.) More pervasively, the logistical chaos caused by intermittent evictions from homeless encampments and confiscation of possessions contributed to missing outpatient appointments. More subtly, purposefully antagonistic treatment by law enforcement officers also set the tone for hostility and distrust in interactions with all state-funded service institutions, especially on the part of African Americans who subscribed to the outlaw persona. We observed greater and more antagonistic police surveillance of African Americans than whites. African Americans sometimes responded oppositionally to law enforcement, resulting in escalated cycles of negative contact. For example, African Americans were more likely than whites to protest verbally when ticketed and to drink alcohol in public without hiding their bottles in a paper bag. They also more frequently displayed the intoxicating effects of their heroin consumption (“nodded out”) in public venues.

We consulted California court records to verify the lifetime adult incarceration self-reports of our ethnographic respondents. Everyone had been incarcerated more than once, but the African Americans had spent more time in prison over their lifetimes. We identified two questions on the epidemiological survey that addressed socialization into crime, and both support the ethnographic data. Among the older (≥ 45 y) injectors, a higher proportion of African Americans reported having been incarcerated during their lifetimes (71% versus 57%; *p* = 0.002) and the median number of years spent in prison (among those who had at least one week of incarceration) was higher for African Americans (10 y versus 7 y; *p* = 0.002). This pattern also held for younger injectors, except that the difference in median years of incarceration was not statistically significant (Table 1).

### Ethnic Patterns to Polydrug Consumption and Modes of Injection

Both the whites and African Americans in our ethnographic social network sample explicitly identified themselves as “heroin addicts.” Once their physical and psychological craving for heroin was satisfied, however, they consumed different psychoactive substances. The whites tended to buy inexpensive fortified wine (primarily Cisco Berry brand) with their extra money, while the African Americans usually sought crack (cocaine hydrochloride converted into its base, i.e., smokeable form). Many of the whites drank heavily and often fell asleep around sunset, while the African Americans frequently stayed up through the night smoking crack. Most of the whites smoked crack occasionally when the opportunity presented itself, but—with a few notable exceptions—they did not actively seek it out. Furthermore, whites who smoked crack intensively were criticized by other whites in explicitly racist language for “behaving like a n…[expletive deleted].”

Both the whites and African Americans said they preferred to inject heroin intravenously rather than subcutaneously or intramuscularly, because of the initial “rush” of pleasure provided by a direct dose of opiates into a vein. Their veins, however, were scarred by lifetime careers of daily multiple injections of heroin. All the whites claimed that this scarring made it difficult for them to locate a vein. They often administered their injections subcutaneously into body fat (see again photo by JS at http://www.publicanthropology.org/Photogallery/B&S-Mindy&Petey.htm), sometimes directly through their clothes, without seeking a vein. Consistent with their overall sense of failure as outcasts, many of the whites claimed that they had given up pursuit of the intravenous rush of intoxication; they also relied on methadone treatment and/or detoxification programs more frequently. They claimed not to be able to “get high” from heroin any more.

In contrast, the African Americans in our social network most often managed to find a vein in which to inject heroin intravenously. As part of their socially constructed identity, they actively pursued an ecstatic rush of intoxication. Unlike the whites, they did not conceive of themselves as passively staving off opiate withdrawal symptoms. African Americans, consequently, took more time, often over 20 minutes to administer their intravenous injections with determined effort. In an often visibly bloody procedure they repeatedly probed with their needles, sometimes seeking dangerous injection sites such as the jugular (see photo by JS at http://www.publicanthropology.org/Photogallery/B&S-Hank-Fixes-Jesse.htm) or brachial veins. Similarly, when they generated windfall income, they sometimes celebrated by injecting a mixture of heroin and cocaine (known in street parlance as a “speedball” despite containing no “speed,” i.e., methamphetamine) [49]. Consistent with the known risks of subcutaneous heroin injection, the whites suffered more abscesses than the African Americans [50–52]. The photographic data were especially useful for documenting ethnic distinctions with respect to injection techniques as well as abscesses.

Five questions from the epidemiological survey directly addressed the ethnic components of drug consumption patterns outlined above: Older African Americans reported having smoked crack on a median of 10 days in the past 30 days as compared with 4 days among older whites (*p* = 0.02). Older African Americans also reported significantly more speedball injections (53% versus 41%; *p* = 0.005) and marginally (although not significantly) more cocaine injections (19% versus 16%; ns). They were also significantly less likely to report methadone use in the past year (24% versus 43%; *p* < 0.001) or having had an abscess (44% versus 54%; *p* = 0.03) in the past year. Among younger injectors, similar differences between African Americans and whites were found in crack smoking, injecting speedballs, and having abscesses, but the difference in methadone use was not significant (Table 1), and cocaine injection was more common among whites than African Americans (31% versus 15%; *p* = 0.001).

We expected the difference in the prevalence of abscesses between African Americans and whites to be larger given our strong ethnographic findings on ethnically distinct techniques of administering heroin injections. Socially desirable reporting should, if anything, have further accentuated this difference, since all the African Americans in our ethnographic sample were embarrassed when they had an abscess. To explore this further, five of our 12 strategically targeted follow-up case study interviews were directed at African American injectors who reported having abscesses in the quantitative study. Without being told of our hypothesis, three of the respondents spontaneously described their exceptionalism and two referred specifically to transgressing ethnic norms. One explained that he reported his identity as “black” on forms, but was actually “half Japanese.” He had a white “running partner” with whom he regularly injected subcutaneously. Another, who had self-identified as African American on the questionnaire, specified that he was “really Puerto Rican” and had a white, gay partner. The third defensively dismissed his abscess as a “missed speedball injection” and claimed it was the first soft tissue infection in his long career of injection drug use.

## Discussion

Our cross-methodological inquiry found substantial agreement between the ethnographic and epidemiological data. Among older injectors, statistically significant differences were found in nine of the 14 epidemiological variables that our ethnographic data and clinical observations predicted would differ between older African Americans and older whites. There were some discrepancies, however, between the ethnographic and epidemiological data. On five of the 14 variables older African Americans and older whites did not differ significantly (cocaine injection and income from: job, illegal sources, social services, and family/friends), contrary to what we expected from our ethnographic data. Furthermore, in several of the variables that differed significantly, the differences were considerably smaller than anticipated.

Some of these differences between the ethnographic and epidemiological data are worthy of interpretation. The unexpected lack of difference with respect to cocaine injection may be due to the fact that the UHS samples a wider range of white social networks in which cocaine is more prevalent than in our ethnographic sample, such as among sex workers or among higher status street injectors. The lack of statistical significance for most of the income variables, on the other hand, may simply be due to recall error and/or to social desirability bias, especially when reporting criminal versus legal income in a formal, face-to-face interview. Most importantly, the epidemiological data indicate that in everyday practice individuals often violated the “ethnic ideal types” that we identified ethnographically. The small differences between African Americans and whites on several of the variables demonstrate the risk of stereotyping racial and cultural categories. For example, even though older African Americans were significantly less likely to report income from panhandling, a practice they considered low in prestige, 17% still reported that source of income. Similarly, although we never observed older whites purposefully injecting speedballs during our decade of ethnographic fieldwork, 41% of whites on the epidemiological survey reported having injected a speedball in the last 30 days. Furthermore, even when distinctions were substantial between ethnic groups on a variable, diversity around those distinctions existed among individual members within the same ethnic group.

Our ethnographic data suggest that the meaning of ethnicity is affected by “social structural” forces such as the existence of youth gangs, the disappearance of industrial jobs, the segregation of neighborhoods, and the organization of families, which in turn are also affected by public policies such as law enforcement, public education, or job training. The congruence of these macro-power vectors may explain the generational ethnic patterns in drug use and homelessness that have been documented by ethnographers [24,27,53], epidemiologists [21,54,55], and historians [23]. For example, the outlaw persona that most of the older African Americans in our ethnographic network projected can be understood as a specific relationship to a definition of masculine dignity that was persuasive for a historical cohort of poorly educated, young inner city men from working-class families headed by parents who were rural immigrants fleeing the economic servitude and racist legislation of the Deep South [56,57]. This older generation of African American heroin addicts came of age in San Francisco in the 1960s and 70s when heroin was fashionable and readily available to street-based youth who celebrated an oppositional outlaw identity. The loss of unionized jobs for high school drop-outs simultaneous with the rise of segregated youth gangs in Californian inner cities that excluded whites in the mid-1970s and decreased funding for public schools in impoverished neighborhoods resulted in the disproportionate incarceration of African American teenagers for gang fighting. This pattern is consistent with national statistics revealing that urban African American and Latino communities experienced the brunt of the expansion of incarceration rates under the prosecution of the War on Drugs [58]. In this institutional and political economic context, masculine models of achievement among a subgroup of poor African American youth may have shifted away from the legal manual labor employment that their parents had actively sought.

### Clinical Implications

Clinically oriented ethnographic research provides rich contextual data that can help explain problems and paradoxes affecting the health and health care of populations. It can also offer interpretations of epidemiological data as well as socially plausible causal explanations for associations. Bringing quantitative and qualitative perspectives into conversation among researchers collaborating across disciplines has the potential to create an analysis that is richer than the sum of its parts, especially for clinicians who rely on quantitative evidence-based data, but whose practices include diverse patients with complex case histories [59,60].

Understanding patients—their histories, perspectives, and expectations—enables successful clinical encounters. The anthropological technique of cultural relativism, which is distinguished by suspension of moral judgment, self-reflection on biases, and an attempt to see the clinical encounter through the eyes of oppositional patients is key to this endeavor. Clinical practices and protocols can benefit from an understanding of how risk behavior and resistance to authority are ethnically scripted. When we accompanied injectors to the county hospital we sometimes observed confrontational interactions with hospital staff and medical practitioners. If oppositional behavior is taken at face value and is treated as a personal, racial, or fixed cultural characteristic, health care providers may define patients as belligerent and cease attempting to deliver optimal care. For example, behaviors that are seen as unacceptable in the clinic, such as cursing, shouting, threatening, and acting angry may be seen by the patient as functional and respected—even dignified—ways of asserting one's rights, self-control, and intelligence on the street. Clinicians who recognize the “outlaw” and “outcast” as socially determined personae, and who understand the dramatic social structural vulnerabilities that can prevent patients from interacting effectively in health care settings, may be able to engage more productively with these challenging and nonadherent patients. Effective clinicians set clear, explicit expectations and respond consistently to behaviors that violate those expectations, without terminating or withholding care unless absolutely necessary [34,61]. (See also pp. 164–167 in [61].)

One under-reported aspect of culturally competent care potentially benefits clinicians because more understanding may cause less frustration. In our observations of clinical interactions, physicians often reported encounters as frustrating even when the street-based injector felt that it was positive. On several occasions exhausted medical residents who were well liked by our participants broke down when describing their cases to us. They interpreted the oppositional and nonadherent behavior of their patients as a personal affront. Disengaging from this sense of personal betrayal and/or failure is key to improving the clinical relationship with challenging populations. Furthermore, we found that even when behaving negatively in the clinical encounter, street-based injectors often wanted more care but faced logistical challenges to adhering to medical instructions due to their precarious physical living conditions (see photo by JS at http://www.publicanthropology.org/Photogallery/B&SFelix-recuperates.htm). The most frequent complaints to us by older injectors revolved around early release from inpatient care and refusal of admission to emergency care. (The patient shown in the intensive care unit photo by JS at http://www.publicanthropology.org/Photogallery/B&S-Jesse,Hank,Petey.htm spent six weeks in the hospital recovering from hepatic failure. The day before falling unconscious, he insisted that he was not “sick enough” to warrant admission when the photoethnographer offered to drive him to the emergency room. This photograph also reveals a cross-ethnic expression of solidarity and emotional vulnerability in the safety of the intensive care unit.) In sum, a culturally and institutionally competent understanding of ethnic populations of drug injectors may reduce difficult clinical interactions and resultant physician frustration while improving patient access and adherence to care—and diminish extreme forms of suffering.

Two of the epidemiological variables we used to explore ethnic distinctions directly measured health outcome and clinical care delivery—self-reported abscesses and receipt of methadone treatment. Consistent with national epidemiological data [62], our methadone findings suggest that institutional barriers blocking African American access to methadone treatment need to be addressed. Our ethnographic findings confirm the anthropological literature from across the US suggesting that methadone clinics are organized in a manner that is overbearing and repressive to many patients. Some injectors, especially self-respecting outlaws, consider methadone maintenance to be a badge of shame [63–66]. (We did not include HCV and HIV infection as variables for ethnic comparison, because HCV is highly infectious and exceedingly prevalent in our population. HIV, in contrast, has a relatively low prevalence and responds to many confounding behavioral and structural risks. Every member of our ethnographic network was HCV positive, compared to 85.1% of the larger UHS sample of men who inject heroin. Only one member of our ethnographic sample was HIV positive, and he was the only man to report having sex with men. Overall HIV prevalence in the larger UHS sample of men was 9.1%.)

Ethnic differences in injection practices may explain the higher rates of abscesses among white injectors, but they do not sanction ethnic-specific clinical and preventive practices. Clinicians can engage effectively with injectors of any ethnicity by discussing practices that promote vein longevity [67,68] and that reduce bacterial and viral infection risks [69,70]. (See again photos by JS at http://www.publicanthropology.org/Photogallery/B&S-Sid-in-Hole.htm and http://www.publicanthropology.org/Photogallery/B&S-Mindy&Petey.htm, to note the practical challenges to adhering to hygienic injection practices on the street.) On an institutional level, outpatient wound clinics specializing in management of injection-related complications in a nonjudgmental manner can provide a high level of care at less expense than the inpatient and emergency units at which most injectors access abscess care [32,71]. Similarly, needle exchanges and clinics tailored for specific categories of injectors such as youth, women, sex workers, or men who have sex with men can bring especially high-risk drug users into regular contact with treatment and prevention services.

Public health institutions and clinicians are in an effective position to take more leadership to reduce the unintended negative consequences of law enforcement practices on the health of street-based populations. The efforts of clinicians and public health outreach workers are too often directly contradicted by police in the very same community—especially when officers aggressively confiscate the possessions of street people and repeatedly search suspected addicts for syringe possession [72–78]. Addressing the unintended health consequences of law enforcement at both the policy and the community level is especially important for African American injectors who are in disproportionately negative contact with the police. More broadly, regardless of the ethnicity of patients, clinical and public health initiatives are most successful when they lower barriers to care by engaging drug injectors nonjudgmentally around risk reduction to prevent the harm associated with drug use. These approaches do not necessarily demand abstinence as a prerequisite for accessing services and are generally referred to as “risk reduction” or “harm reduction” (see, for example, the World Health Organization definition of the term [79]).

### Limitations

Self-report distortion with respect to sex, drugs, and crime is well documented in both the ethnographic [49,59] and epidemiological literatures [80–84]. An NIH/National Institute on Drug Abuse-funded cooperative agreement, for example, documented validity and test-retest reliability for injection drug use data, but low reliability for the details of needle sharing practices [85–87] The challenge posed by socially desirable responses is not limited to quantitative data. All of the older injectors had received public health counseling on numerous occasions and knew the details of HIV transmission risk through injection practices. In the shooting encampments, for example, we photographed addicts engaging in unsanitary sharing of paraphernalia at the same instant that we tape recorded them asserting their commitment never to share paraphernalia.

Our ethnographic research participants do not represent the range of street-based injection drug users in San Francisco, because we purposefully limited our sample to one social network of older heroin injectors to document risk behavior through participant observation in a natural setting. Our qualitative data benefit from being put in quantitative context within the large UHS epidemiological database, but their generalizability remains limited. Ethnographic methods are subjective by definition. An ethnographer's personality and theoretical orientation as well as the serendipity of everyday social interaction affect access to participant-observation data.

Our epidemiological data benefited from 17 years of community-based sampling and interviewing by an experienced staff. Targeted sampling avoids the selection bias inherent in recruiting drug users from institutions such as clinics, emergency rooms, substance abuse treatment facilities, or the criminal justice system. It is not possible, however, to obtain a definitively representative sample of hidden populations who engage in illegal drug use. Our street-based recruitment disproportionately sampled injectors who were able to participate in study procedures during daytime hours; those who wanted and needed the nominal stipends we offered; those who were willing to identify themselves to study staff (and other study participants in their community) as illicit drug injectors; and those who liked their previous experiences when participating in the UHS. Finally, age effects are part of the African American versus white differences we are documenting epidemiologically, and they may also confound the ethnic categories, because African Americans had a higher median age than whites in all quartiles (49 years [interquartile range 45–53] versus 40 years [interquartile range 33–47]).

### Conclusion: Replacing Race with Ethnicity

Merging epidemiological, clinical, and ethnographic data demonstrates the importance of understanding ethnicity as a product of social and historical configurations. An individual's relationship to ethnic ways of being in the world is fluid and changes over time. It is shaped by identifiable social structural forces including immigration patterns, labor market segmentation, and social and spatial interaction. The variable “race” that is collected on all NIH-funded studies is more usefully understood as a socially constructed form of ethnicity that is specific to the US, where skin color imposes an experience of racism at the everyday level which sometimes results in distinct patterns of behaviors, values, and demographic characteristics.

Clinical cultural competence requires an awareness of the social embeddedness of individuals and populations. Understanding the social and structural forces that affect group identities allows for a better appreciation of the variation in individual behaviors that harm health. It also provides a tool for reducing institutional barriers to optimal care for socially vulnerable populations. Excellent cultural competency requires an excellent evidence base. Sociobehavioral epidemiology needs to expand its database on the social and cultural determinants of health and health disparities, including deeper exploration through multidisciplinary approaches of the underlying reasons for observed associations or differences. Social epidemiology requires not only the usual standard of biological plausibility to identify credible casual associations, but also social plausibility. Providing clinicians and public health advocates with the tools to identify the social and structural logics that produce nonadherent behaviors that appear to be racially inscribed or to be the product of individual malevolence may be a first step toward addressing economic and ethnic disparities in health.

## Supporting Information

Alternative Language Abstract S1 Italian abstract by Alessandro De Giorgi with editing by Stefania De Petris (28 KB DOC)

Alternative Language Abstract S2 Spanish abstract by Fernando Montero Castrillo (27 KB DOC)

Alternative Language Abstract S3 French abstract by Alison Hathaway (29 KB DOC)

Text S1 Sample Consent Form: Clinical (33 KB DOC)

Text S2 Sample Consent Form: Epidemiology (38 KB DOC)

## Abbreviations

HCV: hepatitis C virus
NIH: National Institutes of Health
UHS: Urban Health Study
