# Supplementary material for: Reinterpreting Ethnic Patterns among White and African American Men Who Inject Heroin: A Social Science of Medicine Approach
Source: PLoS Med. 2006 Oct 24;3(10):e452. doi: 10.1371/journal.pmed.0030452 (PMC1621100; doi:10.1371/journal.pmed.0030452)
Supplement: Supplementary file 1 [file pmed.0030452.sd001.doc]

**PER UNA RILETTURA DEI MODELLI ETNICI DI TOSSICODIPENDENZA DI STRADA TRA EROINOMANI BIANCHI E AFRO-AMERICANI:**

**UNA PROSPETTIVA METODOLOGICA INTEGRATA**

**DI SOCIOLOGIA MEDICA**

***ABSTRACT***

**CONTESTO:**

Gli eroinomani di strada rappresentano un gruppo particolarmente esposto a prestazioni sanitarie scadenti e a forme di stigmatizzazione sociale. Un trattamento clinico efficace e un intervento sanitario appropriato richiedono una buona conoscenza dell’ambiente culturale e delle esperienze di queste persone. Le teorie e le metodologie sociologiche offrono strumenti utili per comprendere la logiche della disuguaglianza etnica ed economica che determinano sofferenza individuale e stress istituzionale.

**METODOLOGIE E RISULTATI:**

Abbiamo adottato un approccio metodologico integrato che comprende dati statistici, clinici ed etnografici raccolti nel corso di due studi a lungo termine condotti a San Francisco – il primo di carattere epidemiologico, il secondo etnografico – volti ad analizzare l’impatto della collocazione etnica su eroinomani di sesso maschile e di età pari o superiore a 45 anni, bianchi e afro-americani. I risultati etnografici sono stati incrociati con l’analisi di 14 variabili epidemiologiche rilevanti stratificate per età media e collocazione etnica.

All’interno del campione etnografico di relazioni sociali da noi osservate, abbiamo riscontrato tra gli (auto-identificati) afro-americani e i bianchi alcune differenze significative nelle pratiche sociali relative a 1) consumo di droghe, 2) produzione di reddito, 3) rapporti sociali e istituzionali, 4) salute e igiene personale. Gli afro-americani e i bianchi tendevano infatti a sperimentare relazioni strutturali diversificate a fronte di una comune condizione di dipendenza e povertà. In particolare, questa generazione di tossicodipendenti di San Francisco è figlia degli immigrati poveri che dalle campagne si sono riversati nell’area urbana in una fase (dalla fine degli anni ’60 a tutti gli anni ’70) caratterizzata dalla scomparsa del lavoro industriale e dall’ascesa dell’eroina; si tratta dello stesso periodo in cui hanno preso vita le gang giovanili delle *inner city* segregate e ha avuto inizio la guerra alla droga. Gli afro-americani hanno sperimentato precoci contatti negativi con il sistema punitivo, ma hanno anche mantenuto rapporti di lungo termine con le rispettive famiglie allargate; al contrario, molti bianchi sono stati allontanati dalla famiglia in seguito ai primi reati connessi alle droghe. Queste circostanze storico-strutturali hanno generato differenti presentazioni del sé. I bianchi si rappresentavano come degli emarginati, sconfitti dalla dipendenza; sostenevano di assumere eroina per allontanare il “mal di roba” più che per provare piacere. Al contrario gli afro-americani esibivano la propria dipendenza fisica come una ricerca di autonomia e piacere; si consideravano criminali professionisti e rifiutavano qualsiasi connotazione abietta. In gran parte (ma non senza eccezioni), questi risultati etnografici sono stati confermati dalla nostra base epidemiologica, evidenziando una certa variabilità dei comportamenti all’interno delle diverse categorie etniche.

**CONCLUSIONI:**

L’integrazione tra metodologie e prospettive di carattere qualitativo e quantitativo sottolinea l’esigenza di proiettare la pratica clinica al di là delle semplici categorie razziali o culturali. Un approccio sociologico-clinico permette di comprendere in che modo i processi socio-culturali sono mediati da relazioni di potere storicamente fondate e istituzionalmente consolidate. Il riconoscimento delle logiche che presiedono a modelli etnicamente peculiari di comportamento tra i tossicodipendenti di strada costituisce il presupposto per una competenza culturale e un rapporto clinico efficace. Questo riduce il rischio di un’assistenza medica inadeguata nei confronti di una popolazione di pazienti particolarmente vulnerabile e difficile da trattare. Infine, le prospettive sociologiche possono contribuire a spiegare le più estese disuguaglianze relative alla condizione sanitaria; possono inoltre ispirare nuove iniziative pubbliche di carattere strutturale che consentano agli operatori clinici di assumere un ruolo più attivo nella revisione di politiche sociali negative dal punto di vista della salute.

Abstract translated by Alessandro De Giorgi and edited by Stefania De Petris
