# Supplementary material for: Reinterpreting Ethnic Patterns among White and African American Men Who Inject Heroin: A Social Science of Medicine Approach
Source: PLoS Med. 2006 Oct 24;3(10):e452. doi: 10.1371/journal.pmed.0030452 (PMC1621100; doi:10.1371/journal.pmed.0030452)
Supplement: Supplementary file 2 [file pmed.0030452.sd002.doc]

Los patrones étnicos entre los hombres blancos y afroamericanos que se inyectan heroína en San Francisco, California: Un acercamiento de las ciencias sociales médicas

**SINOPSIS**

**CONTEXTO**

Los usuarios callejeros de heroína inyectada constituyen un grupo poblacional especialmente vulnerable, sujeto a deterioros de salud y estigma social. Para desarrollar un tratamiento clínico efectivo para esta población, así como una intervención en el plano de la salud pública, es necesario comprender sus experiencias y su ambiente cultural. La teoría y los métodos de las ciencias sociales ofrecen herramientas útiles para entender la lógica de las disparidades económicas y étnicas que causan sufrimiento individual y estrés a nivel institucional.

**MÉTODOS Y HALLAZGOS**

Utilizamos un acercamiento multi-metodológico que incorporó los datos clínicos, cuantitativos y etnográficos recolectados por dos estudios contemporáneos—uno epidemiológico y otro etnográfico—que se han estado realizando a largo plazo en San Francisco. De este modo, exploramos el impacto de la etnia en usuarios masculinos de heroína inyectada—hombres blancos y afroamericanos cuya edad iguala o sobrepasa los 45 años de edad. Triangulamos nuestros resultados etnográficos examinando 14 variables epidemiológicas relevantes, ordenadas de acuerdo con la etnia y la edad mediana.

Observamos diferencias significativas entre las prácticas sociales de los hombres que se auto-identifican como afroamericanos y las de aquéllos que se identifican como blancos en nuestra red social. Percibimos estas diferencias con respecto a los patrones de 1) consumo de drogas, 2) generación de ingresos, 3) relaciones sociales e institucionales y 4) higiene personal. Las personas blancas y afroamericanas compartían sus condiciones de pobreza y adicción, pero su relación estructural con tales condiciones era divergente. Específicamente, esta generación de inyectores de San Francisco está compuesta por los hijos de inmigrantes pobres procedentes de zonas rurales. Crecieron en una era (la segunda mitad de los 60 y la década de los 70) en la que los empleos industriales desaparecieron y la heroína cobró popularidad. Ésta también fue la era del auge de las pandillas juveniles en las zonas segregadas urbano-marginales y el comienzo de la Guerra contra las Drogas. Los afroamericanos tuvieron un contacto más temprano y negativo con los ejecutores de la ley, pero mantuvieron lazos de largo plazo con sus familias extendidas. La mayoría de los blancos fueron expulsados de sus familias cuando empezaron a involucrarse en crímenes relacionados con las drogas. Estas condiciones histórico-estructurales generaron distintas presentaciones de sí. Los hombres blancos asumieron el perfil del exiliado, derrotados por la adicción. Decían estar inyectándose heroína no para encontrar placer, sino para aplacar “la enfermedad de las drogas” (“dopesickness”). Los hombres afroamericanos, por el contrario, concibieron su adicción como un acto de oposición—parte de su búsqueda de autonomía y placer. Llegaron a considerarse delincuentes profesionales y a rechazar cualquier semblante de abyección. Muchos, pero no todos, de estos hallazgos etnográficos fueron corroborados por nuestros datos epidemiológicos, poniendo de relieve la variabilidad de los comportamientos en el interior de las categorías étnicas.

**CONCLUSIÓN**

Una conversación entre las metodologías y perspectivas cuantitativas y cualitativas pone de manifiesto que la práctica clínica debe ir más allá de las simples categorías raciales y culturales. Un método científico social-médico permite vislumbrar de qué manera los procesos socio-culturales son mediados por las relaciones de poder históricamente enraizadas e institucionalmente ejecutadas. Reconocer la lógica de los patrones étnico-específicos de comportamiento entre los inyectores callejeros es el cimiento de la competencia cultural y de las relaciones clínicas exitosas. Reduce el riesgo de un cuidado médico sub-óptimo para una población de pacientes excepcionalmente vulnerable y desafiante. Los métodos científico-sociales también pueden ayudar a explicar las desigualdades de salud de mayor escala e informar nuevas maneras de abordar las iniciativas de salud pública en el plano estructural, para así permitirles a los clínicos tomar un rol de liderazgo en la transformación de las políticas sociales que tienen consecuencias negativas para la salud.
