# Supplementary material for: Reinterpreting Ethnic Patterns among White and African American Men Who Inject Heroin: A Social Science of Medicine Approach
Source: PLoS Med. 2006 Oct 24;3(10):e452. doi: 10.1371/journal.pmed.0030452 (PMC1621100; doi:10.1371/journal.pmed.0030452)
Supplement: Supplementary file 3 [file pmed.0030452.sd003.doc]

Résumé

Relations ethniques entre les hommes blancs et afro-américains qui s’injectent héroïne dans les rues de San Francisco, Californie: Une approche des sciences sociales en médecine

**Contexte**

Les consommateurs d’héroïne représentent une population particulièrement vulnérable, qui souffre de mauvaise santé et de stigmatisation sociale. Pour développer un traitement clinique et une santé publique efficace pour servir cette population, il faut comprendre leur milieu culturel et leurs expériences quotidiens. Les outils théoriques et méthodologiques des sciences sociales facilitent une compréhension des logiques sociales qui causent les disparités dans les conditions médicales entre classes socio-économiques et entre groupes ethniques qui font souffrir les individus et déséquilibrent les institutions.

**Méthodes et Résultats**

Nous avons utilisé une approche multi méthodologique qui consiste de donnés quantitatifs, cliniques, et ethnographiques recueillit par deux études contemporaines de longue duré, un épidémiologique et un ethnographique, pour explorer l’impact de l’ethnicité des hommes afro-américain ou blanc âgés de plus de 45 ans qui s’injectent l’héroïne dans la rue. Nous avons triangulé nos conclusions ethnographiques avec 14 variables epidemiologiques pertinent stratifié par âge médian (45) et par ethnicité.

Nous avons observé des différences significatives dans les pratiques sociales entre afro-américains et blancs dans le réseau social que nous avons étudié ethnographiquement en ce qui concerne les modèles de 1) consommation des drogues, 2) génération de revenu, 3) rapports sociales et institutionnels; 4) santé et hygiène personnelles. Les afro-américains et les blancs avaient tendance à avoir des rapports différents avec leurs dépendances aux drogues et leur situation d’exclusion sociale et pauvreté. Les consommateurs d’héroïne à San Francisco âgé de plus de 45 ans font parti d’une seconde génération d’immigrés urbains d’origine rurale pauvre. Pendant la fin des années 60 aux années 70, les emploies industriels ont disparu et l'héroïne est devenue à la mode. C’était aussi l’époque d’augmentation des gangs adolescents ségrége ethniquement (à l’exclusion des jeunes blancs) et aussi l’amorce de la guerre a la drogue du gouvernement fédérale. Les afro-américains ont eu alors des contacts plus tôt et plus negatif que les blancs avec la police. Ils ont aussi maintenu des liens à longs termes avec leurs familles. La plupart des blancs ont été expulsées de leurs familles quand ils ont commencé à s’injecter l’héroïne. Ces conditions historique-structurales familiales ont produit des présentations distinctes de «persona social». Les blancs se sont dénommés comme parias. Ils se conçoivent comme «malade» à cause de leur dépendance sur les drogues et l’alcool et disent qu’ils ne cherchent plus le plaisir de la drogue. Ils fuient seulement la douleur d’être en manque d’opiacé. Par opposition aux blancs, les afro-américains poursuivent le plaisir et l’autonomie dans leur prisent de drogues. Ils se considèrent comme des hors-la-loi professionnels et rejètent l’apparence d'abjection. La plupart de ces résultats ethnographiques ont été corroborés par notre base épidémiologique, mais pas tons, et cela confirme la variabilité des comportements dans les catégories ethniques.

**Conclusion**

L’intégration des méthodologies et des perspectives quantitatives et qualitatives démontre le fait que la pratique clinique doit dépasser des catégories raciales ou catégories culturelles simples. Une approche des sciences sociales fournit une compréhension des processus socioculturels qui sont généré par des relations historiques et institutionnels. Un concept des modèles comportementaux ethnique-spécifiques des consommateurs de rue forme la base pour une compétence culturelle et pour le développement de meilleurs rapports dans les services cliniques. Cette reconnaissance réduite le risque de mal soigner une population particulièrement vulnérable et difficile. Les études de science sociale expliquent à plus grande échelle les logiques sociales pour les inégalités et orientent des nouvelles initiatives de santé publique vers le niveau structurel et institutionnel et permettrent aux secteur médicale de s’engager a changer les politiques sociales qui ont des conséquences négatives sur la santé.

Translated by Alison Hathaway.
