# Supplementary material for: Reinterpreting Ethnic Patterns among White and African American Men Who Inject Heroin: A Social Science of Medicine Approach
Source: PLoS Med. 2006 Oct 24;3(10):e452. doi: 10.1371/journal.pmed.0030452 (PMC1621100; doi:10.1371/journal.pmed.0030452)
Supplement: Supplementary file 4 [file pmed.0030452.sd004.doc]

## Differential Health Risks Among Substance Abusers

# UNIVERSITY OF CALIFORNIA, SAN FRANCISCO

**CONSENT TO BE A RESEARCH SUBJECT**

**A. PURPOSE AND BACKGROUND**

Philippe Bourgois, PhD, Chair of the Department of Anthropology, History and Social Medicine, is conducting a research study to help understand the experience of socially vulnerable people who receive health care. The study is being funded by the National Institutes of Health. You are being asked to participate in this study because of your involvement in receiving and/or providing health care. We are documenting the daily experience of being at risk of HIV (and/or other medical conditions) and/or living with HIV (and/or other medical conditions) and/or working with people infected by HIV (and/or affected by other medical conditions).

**B. PROCEDURES**

1) If you agree to be in this study the following will occur: Philippe Bourgois or one of the other researchers participating in this study will spend time with you and talk to you about your everyday life. If you agree some of these conversations will be tape-recorded and with your permission you may also be photographed and videotaped.

2) Participation in the study may take a great deal of your time. We cannot estimate a total number of hours with any precision as the project is scheduled to last for at least another three years, and with your permission Bourgois or one of his assistants will be visiting with you on a regular basis. Sometimes one of us may accompany you during the day or evening.

3) Some of our conversations will include discussions of your sexual activity, drug and alcohol use, illegal income generating strategies, and past experiences of violence and child abuse.

**C. RISKS OR DISCOMFORTS**

1) Some of the conversation topics might make you uncomfortable or upset but you are free to decline to answer any questions or to stop continuing to participate in the conversation whenever you are not comfortable with the subject. You are free to ask any of the researchers to leave or stop talking at any time.

2) Confidentiality: The researchers will keep information about you as confidential as possible, but complete confidentiality cannot be guaranteed. On rare occasions research records have been subpoenaed. The National Institutes on Health has given the researchers on this project a Federal Certificate of Confidentiality which says that courts cannot force the researchers to reveal information about your participation in the study. Your names will be coded in all our transcriptions and fieldwork notes. Only Dr. Bourgois and the members of this research team will have access to your coded study records, audiotapes, videotapes or photographs. Some of the members of his team may include your health care providers or may be your supervisor or employer. When the study is finished the audiotapes and videotapes will be stored in a secure, locked archive with identifying markers removed from the tapes. No individual identities will be used in any reports or publications that may result from this study. We will avoid making you fully identifiable on the videotapes or photographs and we will avoid rendering identifiable the locations where we film. It is impossible, however, to ensure anonymity on the videos. Edited excerpts will be used for academic and public health presentations, including training videos for healthcare providers, outreach workers and scholarly presentations at research conferences and public health research centers and in scientific publications.

3) There is a risk that one of your medical providers and/or supervisors/employers who is part of the research team may recognize you, despite your coded name. However, the medical providers and/or supervisors/employers working on this project have committed themselves to maintaining your anonymity and to not using the information they may recognize in the data to your or anyone’s disadvantage. Despite this commitment, there is always a risk that their opinions of you may be affected and this may affect your treatment.

**D. BENEFITS**

There will be no direct benefit to you for participating in this study. However, the information that you provide may help health professionals better understand how to develop outreach programs to serve HIV positive people and help them stay healthy.

**E. COSTS**

There will be no costs to you as a result of taking part in this study.

**F. PAYMENT**

You will not be paid for participating in this study.

**G. TREATMENT AND COMPENSATION FOR INJURY**

If you are injured as a result of being in this study, treatment will be available. The costs of such treatment may be covered by the University of California, depending on a number of factors. The University does not normally provide any other form of compensation for injury. For further information about this, you may call the office of the Committee on Human Research at (415) 476-1814.

**H. QUESTIONS**

You have talked to Dr. Bourgois or the person who signed below about this study and have had your questions answered. If you have further questions, you may call him/her at (415) 502-8400.

If you have any comments or concerns about participation in this study, you should first talk with the researchers. If for some reason you do not wish to do this, you may contact the Committee on Human Research, which is concerned with the protection of volunteers in research projects. You may reach the committee office between 8:00 and 5:00, Monday through Friday, by calling (415) 476-1814, or by writing: Committee on Human Research, Box 0962, University of California, San Francisco/San Francisco, CA 94143.

**H. CONSENT**

You will be given a copy of this consent form to keep.

PARTICIPATION IN RESEARCH IS VOLUNTARY. You are free to decline to be in this study, or to withdraw from it at any point. Your decision as to whether or not to participate in this study will have no influence on your present or future status at UCSF. IF YOU AGREE TO PARTICIPATE YOU SHOULD SIGN BELOW:

________________	___________________________________

Date	Signature of Study Participant

_______________	_________________________________­__

Date	Signature of Person Obtaining Consent
