# Supplementary material for: Reinterpreting Ethnic Patterns among White and African American Men Who Inject Heroin: A Social Science of Medicine Approach
Source: PLoS Med. 2006 Oct 24;3(10):e452. doi: 10.1371/journal.pmed.0030452 (PMC1621100; doi:10.1371/journal.pmed.0030452)
Supplement: Supplementary file 5 [file pmed.0030452.sd005.doc]

# Differential Health Risks Among Substance Abusers

# UNIVERSITY OF CALIFORNIA, SAN FRANCISCO

**CONSENT TO BE A RESEARCH SUBJECT**

**A. PURPOSE AND BACKGROUND**

Philippe Bourgois, PhD, Chair of Department of Anthropology, History and Social Medicine, is conducting a research study to help understand the lives of people on the street. The study is being funded by the National Institute on Drug Abuse. You are being asked to participate in this study because you have a great deal of experience with drugs and with life on the street. You are also a member of a social network we have befriended and which we want to document as a special case-study of how street addicts survive from day to day. We want to document the health risks you take, especially with respect to HIV, hepatitis C, and/or abscesses.

**B. PROCEDURES**

1) If you agree to be in this study the following will occur: Philippe Bourgois or one of the other researchers participating in this study will spend time with you and talk to you about your everyday life. If you agree some of these conversations will be tape-recorded and with your permission you may also be photographed and videotaped.

2) Participation in the study may take a great deal of your time. We cannot estimate a total number of hours with any precision as the project is scheduled to last for at least another three years, and with your permission Bourgois or one of his assistants will be visiting with you on a regular basis. Sometimes one of us may even spend the night in your encampment or accompany you during your work day.

3) Some of our conversations will include discussions of your sexual activity, drug and alcohol use, illegal income generating strategies, schooling, and past experiences of violence and child abuse. 4) Today, if you agree, we are going to take you to another study site for HIV and/or hepatitis C testing. The testing will be done by either 1) an independent research organization known as RTI International (directed by the former Head of the Urban Health Study at the University of California, San Francisco) which is interviewing street-based drug users about their health risks; 2) the UFO Study at the University of California, San Francisco which is interviewing youth about hepatitis and other injection-related health risks; or 3) the Medication Adherence Study at the University of California, San Francisco which is interviewing people about the experience of taking HIV medications. They will counsel you on the meaning of the blood test for HIV or hepatitis C before you consent to be tested. If you consent you will be interviewed and tested. They will describe all of the procedures in detail and give you appointments to receive the results of your tests in person and you will be counseled about the meaning of those results before and after the test. If you agree to be tested they will have you sign their consent form and explain it to you.

**C. RISKS OR DISCOMFORTS**

1) Some of the conversation topics might make you uncomfortable or upset but you are free to decline to answer any questions or to stop continuing to participate in the conversation whenever you are not comfortable with the subject. You are free to leave the conversation at any time, or to ask any of the researchers to leave or stop talking at any time.

2) Confidentiality: Participation in research will involve a loss of privacy, however, your records will be handled as confidentially as possible, especially your HIV and/or Hepatitis C test results. A positive HIV and/or Hepatitis C test will not be reported. In this study you will be asked about drug and other possibly illegal activities. The researchers will keep information about you as confidential as possible, but complete confidentiality cannot be guaranteed. On rare occasions research records have been subpoenaed. The National Institute on Drug Abuse has given the researchers on this project a Federal Certificate of Confidentiality which says that courts cannot force the researchers to reveal information about your participation in the study. No individual identities or descriptions will be used in any reports or publications from this study and your name will not be recorded at the time of the test. In our other research records, your name will be coded in all our transcriptions and fieldwork notes. Only Dr. Bourgois and the members of this research team will have access to your coded study records and audio and videotapes. When the study is finished the audio-tapes and videotapes will be stored in a secure, locked archive with identifying markers removed from the tapes. No individual identities will be used in any reports or publications that may result from this study. We will avoid making you fully identifiable on the videotapes and we will avoid rendering the locations where we film identifiable as well. It is impossible, however, to insure anonymity on the videos. They will be used primarily by our research team for analyzing the micro-details of injection processes and other activities of daily life. Edited excerpts will be used for academic and public health presentations, including training videos for outreach workers and scholarly presentations at research conferences and public health research centers, and scientific publications.

3) You may feel uncomfortable answering some of the personal questions they ask you but you may refuse to answer any questions.

4) Drawing blood may hurt a little and could bruise.

5) Being tested for HIV and/or for Hepatitis C may cause anxiety regardless of the test results. A positive test means that you have been infected with the HIV and/or Hepatitis C virus, but no one can say for certain when, if ever, you will become sick with AIDS and/or liver disease or a related condition. Receiving positive results may make you very upset. If other people learn about your positive test results, you may have trouble obtaining insurance or employment. If your test is negative, there is still the possibility that you could be infected with the HIV and/or the Hepatitis C virus and test positive at some time in the future. There is always the possibility that the test results could be wrong.

**D. BENEFITS**

There will be no direct benefit to you for participating in this study. However, the information that you provide may help health professionals better understand how to develop outreach programs to help street addicts avoid HIV, Hepatitis, and stay healthy. If you agree to be tested for HIV and/or Hepatitis C and your test results are negative you may feel relieved. If your tests are positive you will be alerted to protect your sexual or drug-using partners from infection and you will be referred for a medical follow-up exam.

**E. COSTS**

There will be no costs to you as a result of taking part in this study.

**F. PAYMENT**

You will be paid $10 cash when you go to get blood tested. The organization testing you will give you another $10 cash when they draw your blood and conduct their interview. They will also pay you $10 cash when you return to obtain your HIV and/or Hepatitis C blood test results.

**G. TREATMENT AND COMPENSATION FOR INJURY**

If you are injured as a result of being in this study, treatment will be available. The costs of such treatment may be covered by the University of California, depending on a number of factors. The University does not normally provide any other form of compensation for injury. For further information about this, you may call the office of the Committee on Human Research at (415) 476-1814.

**H. QUESTIONS**

You have talked to Bourgois or the person who signed below about this study and have had your questions answered. If you have further questions, you may call him/her at (415) 502-8400.

If you have any comments or concerns about participation in this study, you should first talk with the researchers. If for some reason you do not wish to do this, you may contact the Committee on Human Research, which is concerned with the protection of volunteers in research projects. You may reach the committee office between 8:00 and 5:00, Monday through Friday, by calling (415) 476-1814, or by writing: Committee on Human Research, Box 0962, University of California, San Francisco/San Francisco, CA 94143.

**H. CONSENT**

You will be given a copy of this consent form to keep.

PARTICIPATION IN RESEARCH IS VOLUNTARY. You are free to decline to be in this study, or to withdraw from it at any point. Your decision as to whether or not to participate in this study will have no influence on your present or future status at the University of California, San Francisco.

If you agree to participate please sign below.

________	________________________	__________	___________	____

Date	Signature of Study Participant	*Anonymous identifiers collected by*

*___	____________________*

*epidemiological collaborators*

________	_______________________________

Date	Signature of Person Obtaining Consent
